# Implantable Cardioverter Defibrillators in Prevention of Sudden Cardiac Death in Kidney Transplant Recipients: A Case Series and an Appraisal of Current Evidence

**DOI:** 10.3390/jcm13195820

**Published:** 2024-09-29

**Authors:** Ivana Juric, Lea Katalinic, Vesna Furic-Cunko, Bojan Jelakovic, Nikolina Basic-Jukic

**Affiliations:** 1 Department of Nephrology, Arterial Hypertension, Dialysis and Transplantation, University Hospital Center Zagreb, 10 000 Zagreb, Croatia; lea_katalinic@hotmail.com (L.K.); vfcunko@gmail.com (V.F.-C.); jelakovicbojan@gmail.com (B.J.); nina_basic@net.hr (N.B.-J.); 2 Faculty of Medicine, University of Zagreb, 10 000 Zagreb, Croatia

**Keywords:** sudden cardiac death, implantable cardioverter defibrillator, kidney transplantation

## Abstract

**Background:** Cardiovascular diseases, including sudden cardiac death (SCD), are the leading cause of mortality among kidney transplant recipients (KTRs). While implantable cardioverter defibrillators (ICDs) are established for SCD prevention in the general population, data on the benefits in patients with CKD is scarce and controversial, and there is no established general consensus on their use in this group of patients. Furthermore, data for KTRs are lacking. The aim of this study is to present our experience with ICDs in KTRs and evaluate the outcomes in this population. **Methods:** We retrospectively analyzed medical records of KTRs who received a kidney allograft between October 1973 and December 2023 and received ICDs for the prevention of SCD. **Results:** Of 2282 KTRs, 10 patients (0.44%) underwent an ICD implantation with an average age of 60.6 years at the time of implantation; 9 were male. Primary prevention of SCD was the most common indication, with only one patient receiving an ICD following sudden cardiac arrest. The female patient received an ICD while on dialysis, and the rest of the patients received ICDs in the posttransplant period with an average time of 9.1 years after KT. Kidney allograft function was reduced in all patients at the time of the ICD implantation with an average estimated glomerular filtration rate (eGFR) of 44 mL/min/1.73 m^2^. No ICD-related complications were recorded. Six patients are alive with an average follow-up of 5.2 years. **Conclusions:** ICD implantation in carefully selected KTRs may offer survival benefits and can be a valuable tool in preventing SCD. Larger studies are needed to confirm these findings and establish clear guidelines for ICD use in this specific population.

## 1. Introduction

Kidney transplantation is the best treatment option for patients with end-stage renal disease (ESRD), in terms of better patient survival and quality of life when compared to dialysis [1]. One-year kidney allograft survival exceeds 90% in most transplant centers [2]; however, cardiovascular diseases remain the leading cause of death with a functioning graft. Although kidney transplant recipients (KTRs) have a lower risk of cardiovascular morbidity and mortality compared to dialysis patients, the risk remains significantly higher compared to the general population [3]. Sudden cardiac death (SCD) is a major contributor to mortality among patients with chronic kidney disease (CKD), and the incidence increases with a decrease in GFR, being the highest among patients on dialysis [4,5]. Implantable cardioverter defibrillators (ICDs) are a well-established first-line therapy for the primary and secondary prevention of SCD, significantly decreasing the rate of mortality in the general population [6]. Data on the benefits of ICDs among patients with CKD are scarce and controversial, and consequently, there is no established general consensus on the use of ICDs in this group of patients [7,8]. Furthermore, data for kidney transplant recipients are lacking. The objective of this study is to present our experience with the use of ICDs in KTRs, evaluate their role and outcomes, and provide insights into their potential benefits and limitations in this unique population.

## 2. Materials and Methods

Patients receiving kidney allograft between Oct 1973 and Dec 2023 were prospectively followed over the years. Medical charts and records of kidney transplant recipients who received ICD were retrospectively collected and analyzed. Age, gender, ESRD cause, dialysis vintage, time of transplantation, immunosuppressive protocol, donor type, and concomitant cardiovascular diseases prior transplantation were recorded. Age at the time of ICD placement, time between kidney transplantation and ICD placement, and indication for ICD implantation are recorded. Kidney graft function at the time of ICD implantation, after 6 months, and then, yearly, complications, and outcomes were recorded. Continuous variables were described using mean, minimum, and maximum values, as appropriate.

The study was approved by the Ethics Committee of University Hospital Center Zagreb and performed as a part of the project “Complications of immunosuppressive drugs after kidney transplantation.”

## 3. Results

### 3.1. Patient Characteristics

Of the 2282 patients who received the kidney allograft, 10 patients underwent an ICD implantation (0.44%): 9 males. The cause of ESRD was tubulointerstitial nephritis in 3 patients, and ADPKD in 2 patients; diabetes mellitus, nephroangiosclerosis, and vesicoureteral reflux were the cause of ESRD in one patient each, while in two patients, the cause of ESRD was unknown. The average age at the time of kidney transplantation was 53.0 years (42.7–66.6). Mean dialysis vintage prior to the kidney transplantation was 3.6 years (0.7–8.5). All patients received basiliximab in induction therapy, followed by standard maintenance immunosuppressive therapy. All patients received steroids and CNI; among them, 7 received tacrolimus and 3 cycloporin. A total of 2 patients received mTORi (everolimus), with 1 of them receiving a combination of everolimus and tacrolimus and 1 everolimus and MMF. All patients received at least 3 medications in addition to immunosuppressive therapy (Table 1).

### 3.2. Clinical Characteristics, Indication, Time, Complications, and Outcomes of ICD Implantation

**Figure 1:**
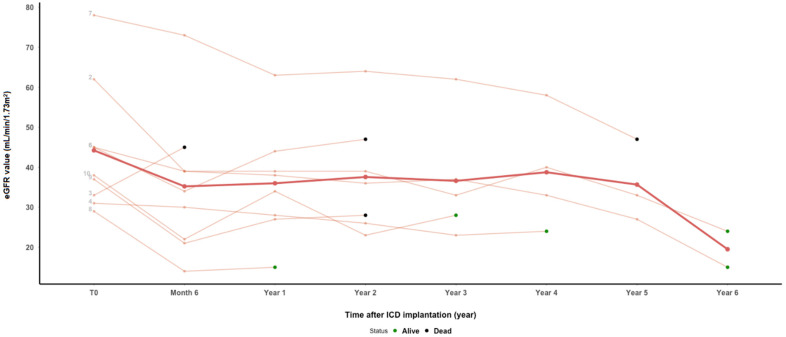
Kidney allograft function following ICD implantation. The bold line: mean eGFR through time (for patients available for analysis at certain follow-up time point).

All male patients underwent the ICD implantation after a kidney transplantation. The mean time of the ICD implantation after the kidney transplantation was 9.1 years (1.8–16.5). The average age at the time of the ICD implantation was 60.6 years (49–69.4). In one patient, the ICD implantation followed a sudden cardiac arrest (SCA), while in all other patients, the ICD implantation was for the primary prevention of SCD. Nine out of ten patients suffered from ischemic cardiomyopathy, and one out of ten from idiopathic dilatative cardiomyopathy. All patients had a history of arterial hypertension prior to the ICD, and four had DM/PTDM. Kidney allograft function was reduced in all patients at the time of the ICD implantation, with an average eGFR of 44 mL/min/1.73 m^2^ (29–78). In a follow-up period, kidney function remained rather stable in all patients (Figure 1). After an average time of 7 years following the ICD implantation (4.5–8.9), three patients, including the female patient who received the ICD prior to a kidney transplantation, experienced end-stage kidney allograft dysfunction and were included in the hemodialysis program. All of these three patients are alive at the time of this report. Four patients experienced cardiovascular complications after the ICD implantation, two patients had a cerebrovascular accident (CVA), one a femoro-femoral bypass, and one patient had an episode of pulmonary edema, while complications related to the ICD were not recorded. After an average of 5.2 (1.6–8.5) years follow-up after the ICD implantation, six patients are alive. Four patients died with time of death ranging from 0.6 to 4.8 years after the ICD implantation (average 2.7) and functioning graft at the time of death. One patient died due to the consequences of severe COVID infection 4.8 years after the ICD implantation at the age of 53.7 years. The second patient with a history of DM and allograft rejection episodes died at the age of 55.8 years due to severe sepsis. The deaths of the remaining two patients were not related to cardiac disease, with the two of them being > 70 years old at the time of death. (Table 1 and Table 2).

The ICD was implanted to the only female patient prior to a kidney transplantation, while in the chronic hemodialysis program, 9 months after the initiation. At the age of 63.4, the CRT-D was placed due to HFrEF with a significantly reduced EF of 20–25% after experiencing a complete AV block. After 4.7 years on hemodialysis and 3.2 years after the CRT-D implantation, she received a kidney allograft. She did not experience cardiovascular events in the posttransplant period or ICD-related complications; however, she was hospitalized multiple times due to non-cardiovascular causes, including recurrent episodes of urinary tract infections and diverticulitis. Kidney allograft function remained rather stable for 4 years after the kidney transplantation, ranging from CKD stage 3a–3b (eGFR 32–46 mL/min/1.73 m^2^), gradually worsening during the 5th posttransplant year, and finally, 5.8 years after transplantation, she developed end-stage kidney allograft failure, and hemodialysis treatment was started. During the posttransplant period, heart function remained stable.

## 4. Discussion

Sudden cardiac death (SCD) is a major cause of death in the general population, accounting for approximately 15% of all deaths and 50% of all cardiovascular deaths [9]. The majority of cases are related to coronary artery disease (CAD), which is attributed to be underlying cause for up to 80% of SCD [10]. The most common mechanism responsible for SCD in this population is ventricular arrhythmia (VA), with VF and VT being the most common type [11]. Patients with CKD are at increased risk of SCD compared to the general population. There is evidence of a reverse correlation between eGFR and the risk of SCD [12]. In a retrospective analysis of the population included in the MADIT-II study, the risk of SCD increased by 17% for every 10 mL/min/min/1.73 m^2^ decrease in eGFR [13]. Similarly, in a large cohort study by Pun et al., a decrease in eGFR was independently associated with SCD with each 10 mL/min decrease in eGFR, increasing the risk of SCD by 11% [14]. Among ESRD patients on dialysis, SCD is a leading cause of death, and it accounts for up to 40% of all-cause mortality [15]. SCD accounts for approximately 15% of all deaths among kidney transplant recipients with a functioning graft. In a study by Marcassi et al. designed to examine the prevalence and factors associated with VA, 30 of 100 patients were found to have VA, the most common arrythmia associated to SCD [16].

ICDs are a recognized and established therapy in the prevention of sudden cardiac death [6]. The beneficial effect of ICD use in the primary prevention of SCD is well-established in large cohort studies, and their impact on the improvement of mortality is confirmed in recent trials. A recent multicentric, prospective, and controlled cohort study EU-CERT-ICD showed the significant survival benefit of ICD use for primary prevention with a 27% lower mortality in the ICD group compared to control in patents with both, ischemic and dilatative cardiomyopathy [17]. In a study by Schrage et al., ICD use in the primary prevention of SCD in heart failure was associated with reduced 1-year and 5-year mortality. However, according to the study findings, only 10% of patients meeting the criteria for primary prevention had an ICD, highlighting their underuse in the primary prevention of SCD [18]. In the DANISH study, the use of ICDs in the primary prevention of patients with non-ischemic heart failure was associated with a reduced rate of SCD. In this study, the use of an ICD was not associated with reduced all-cause mortality, challenging the benefit of ICD use in this group of patients [19]. The beneficial effects of ICD use in secondary prevention are consistent across large trials. According to large meta-analysis, the use of an ICD in secondary prevention is superior to optimal medication therapy and is associated with a 28% reduction in mortality, with the relative risk of arrhythmic death being reduced by 50% in the ICD group [20].

Data on ICD therapy in patients with CKD are scarce and derived from the subanalyses of large ICD clinical trials. In the MADIT-II study, ICD therapy was associated with the survival benefit in patients with eGFR ≥35 mL/min/min/1.73 m^2^ compared to conventional medical therapy, while no significant difference was found in mortality between therapies for eGFR < 35 mL/min/min/1.73 m^2^. Furthermore, ICD treatment was associated with a SCD risk reduction of 66% in patients with eGFR ≥35 mL/min/min/1.73 m^2^, while no significant benefit of ICDs was found in patients with eGFR <35 mL/min/min/1.73 m^2^ [13]. The inferior benefits of ICDs in patients with reduced eGFR (<60 mL/min/min/1.73 m^2^) were confirmed in the sudden cardiac death in heart failure trial (SCD-HeFT) subgroup analysis [21]. In a meta-analysis of 2867 patients previously included in three randomized controlled trials, the benefits of ICDs versus conventional therapy were compared. Survival benefits of ICDs in primary prevention depends on eGFR and is associated with an eGFR ≥60 mL/min/min/1.73 m^2^, while there were no benefits of ICDs among patients with eGFR <60 mL/min/min/1.73 m^2^ [22]. On the other hand, the results of a large meta-analysis of 11 studies, including 20,196 CKD patients, suggest that ICD treatment in patients with eGFR <60 mL/min/min/1.73 m^2^ is associated with survival benefit; however, the benefit was not significant in a subgroup of patients with eGFR <30 mL/min/min/1.73 m^2^ [23]. Current guidelines do not provide clear recommendations regarding the use of ICDs in patients with chronic kidney disease (CKD). Furthermore, existing findings suggest worse outcomes in CKD patients with ICDs compared to patients without CKD; hence, the use of ICDs in these patients remains controversial. Hess et al. conducted a study to examine survival and the factors associated with survival in patients with CKD and ICDs. When compared to patients with no CKD, the risk of death after ICD primary prevention implantation was higher in patients with CKD and proportional to CKD severity. The authors conclude that patients with CKD, even in the advanced stage, should not be excluded as candidates for ICD placement in the primary prevention of SCD. The clinical decision and selection of candidates that would most likely benefit from ICD may be guided and supported by taking into consideration the prognostic factors associated with an increased risk of death [24]. In a meta-analysis by Makki et al., the authors evaluated the impact of ICDs on mortality in CKD patients at high risk of SCD, as well as the effect of CKD on mortality in ICD recipients. CKD patients who received ICDs had a reduced mortality risk compared to those who did not receive ICDs. Furthermore, CKD patients with ICDs had higher mortality then matched ICD recipients without CKD. The authors conclude that the use of ICDs is beneficial in reducing all-cause mortality in CKD patients at high risk of SCD; however, CKD is associated with an increased risk of death [25]. On the other hand, a recent systematic review by Kiage et al. on the use of ICDs in patients with CKD suggests that the routine use of ICDs in patients with CKD may be associated with more adverse outcomes than benefits [26]. In a recently published study, Wright et al. showed that ICD patients with CKD required longer hospitalization and higher treatment costs compared to patients without CKD. Furthermore, CKD was associated with more severe ICD infections and lower survival after the infection [27].

The specific population of KTRs is not addressed in the current guidelines, and the data in the literature are lacking. To the best of our knowledge, this is the first and only series. Despite the high number of transplantations performed over the observed period, only 0.44% of patients had ICDs, reflecting the controversial nature of this important therapeutic measure in this group of patients. Primary prevention was an indication for ICDs in 9 patients, while only 1 patient had an ICD for secondary prevention. Kidney function was reduced in all patients from our cohort at the time of the ICD implantation with average eGFR 44 mL/min/min/1.73 m^2^. The majority of patients had eGFR 30–59 mL/min/1.73 m^2^, 2 patients had eGFR > 60 mL/min/1.73 m^2^, 6 patients, and 1 patient had eGFR < 30 mL/min/1.73 m^2^. Extrapolating data for CKD patients and ICDs in our population, it could be concluded that reduced allograft function may be associated with worse outcomes in KTRs with ICDs. However, based on the small number of patients, it would not be safe to draw this conclusion. Only 1 patient had a rejection episode, and this was the patient who died 1.8 years after the ICD implementation (PT 9, Table 1 and Table 2). These findings may suggest that rejection episodes could be a significant negative predicting factor of the outcomes in kidney transplant recipients with ICDs. The average age of our patients was 60.6 years. As shown in a study by Hess et al., older age was strongly associated with an increased risk of death in patients with CKD and ICDs [24], suggesting that in this group of patients, age along with a life expectancy of 1 year, as recommended in the current guidelines, should be one of the key factors when selecting candidates that could benefit from ICD implementation. The majority of patients from our series were men (9/10). According to the literature, there is a clear disparity between genders regarding the incidence of sudden cardiac death, with men experiencing this condition at a higher rate than woman [28]. Considering the fact that ventricular arrhythmias are the leading cause of SCD in both men and women, the disparity in its occurrence could be attributable to differences in arrhythmogenic sensitivity between genders. However, the mechanisms underlying these differences remain unclear and may stem from a wide range of factors, including hormones, genetic predisposition, and differences in heart structure and function [29]. In a study by Marcassi et al., the majority (77%) of KTRs with recorded VA were male [16].

It remains unclear whether the high rate of SCD in KTRs is associated with the potential arrhythmogenic effects of immunosuppressive drugs. Immunosuppressive drugs may directly increase the risk of arrhythmias by affecting myocardial structural remodeling and intracellular ion transport function. Additionally, they may indirectly influence the risk of arrhythmia by their effects on left ventricular hypertrophy, myocardial fibrosis, hypertension, dyslipidemia, and coronary atherosclerosis [30]. Ikitimur et al. investigated the impact of different immunosuppressive drugs on QT and PR intervals, indicators of increased risk of arrythmias in kidney transplant recipients. The QT interval was prolonged in the posttransplant compared to the pretransplant period in all groups of patients, receiving tacrolimus, cyclosporine, everolimus, and azathioprin, with no difference among the groups. The authors conclude that the prolongation of the QT interval is highly prevalent in kidney transplant recipients receiving different types of immunosuppressives [31]. While there are several reports on CNI-related arrhythmias, there is no strong evidence in larger clinical trials, suggesting the arrhythmic risk is low [32,33,34,35]. There are no published reports indicating that MMF and mTORi increase the risk of arrhythmias. On the contrary, a recent study showed no difference in arrhythmia occurrence in patients treated with everolimus after myocardial infarction [36]. There is evidence that the use of corticosteroids is associated with arrhythmias, including atrial fibrillation, sinus tachy-cardia or bradycardia, premature atrial contractions, and premature ventricular contractions [37]. The majority of our patients received CNI, predominantly tacrolimus, while 2 patients received mTORi. All patients received maintenance steroid therapy, with 1 of them receiving high doses as a part of the allograft rejection treatment. In addition to immunosuppressive therapy, all patients received at least three other medications that could have a direct or indirect proarrhythmic effect through interactions with other medications.

Patients undergoing dialysis can develop CMP, especially uremic CMP, with a decrease in systolic function (HFrEF). The benefit of defibrillator therapy with cardiac resynchronization (CRT) therapy in these patients in the primary prevention of SCD remains controversial, predominantly due to the high risk of death from other non-arrhythmic causes. Furthermore, those patients are very often contraindicated for transplantation due to their higher cardiovascular risk. In a study by Goldenberg et al., 1015 patients receiving CRT with a defibrillator (CRT-D) were divided into two groups based on the stage of CKD. The cumulative incidence of ventricular arrhythmias was 23.5% in patients with CKD stage 1–3a and 12.6% with CKD stage 3b-5 (*p* < 0.001), while the incidence of death not related to arrhythmia was 6.6% and 21.6%, respectively (*p* < 0.001). The authors conclude that patients with advanced CKD and CRT do not seem to benefit significantly from primary prevention ICD due to their relatively low incidence of arrhythmias and rather high non-arrhythmic mortality rate [38]. Our female patient received CRT-D while on dialysis. Successful kidney transplantation was followed by stable cardiovascular status and no cardiovascular events in the early and later posttransplant follow-ups. It could be expected that cardiac function would improve after transplantation due to its cardiovascular benefits as well as beneficial effects on uremic cardiomyopathy. However, this improvement failed to occur, which may be attributed to irreversible cardiac damage, resulting as a consequence of dialysis [39]. Furthermore, kidney allograft function remained relatively stable allowing her 5 years of dialysis-free life. Our experience suggests that carefully selected ESRD dialysis patients and HFrEF may benefit from ICD therapy. Despite the generally high non-arrhythmic mortality rate and lower incidence of ventricular arrhythmias in this population, an individualized assessment could identify those who might derive a significant benefit from ICDs.

Our study has several limitations. The main limitation is the small number of patients. Only 10 (0.44%) of 2282 kidney transplant recipients received ICDs, limiting the generalizability and statistical power to detect significant trends. Second, the study was conducted in a single center, which limits the generalizability and external validation of the findings. The third limitation is the long study period, during which medical practices, including immunosuppressive protocols and ICD technologies, evolved, potentially affecting the consistency of treatment and outcomes. Furthermore, the study was based on retrospectively collected data from medical charts and records, with the possibility of bias and inaccuracies due to incomplete or inconsistent documentation. Finally, there is a significant gender imbalance in favor of male patients, which may affect the applicability of the results to female patients.

## 5. Conclusions

Sudden cardiac death is a significant concern in kidney transplant recipients. Although ICDs are a well-established treatment in prevention of SCD in the general population, data on their use and outcomes in the specific and unique population of KTRs are lacking. Our study suggests that with appropriate selection and management, ICDs could be a valuable tool in preventing SCD in this group of patients. Further research with larger patient cohorts is necessary to tailor and establish clear guidelines and optimize the management of SCD in this unique, vulnerable population.

## Figures and Tables

**Table 1:** Patient characteristics.

PT ID	Gender	ESRD Cause	Age of Tx (Years)	HD Vintage Prior Tx (Years)	Induction IS Therapy	Maintenance IS Therapy	Rejection Episodes	No. of Other Medications > 3
1	f	TIN	66.6	4.7	basiliximab	Tac/MMF/KS	No	Yes
2	m	ADPKD	46.1	1.7	basiliximab	CyA/MMF/KS	No	Yes
3	m	VUR	61.2	1.7	basiliximab	Tac/MMF/KS	No	Yes
4	m	TIN	55.4	8.5	basiliximab	Tac/MMF/KS	No	Yes
5	m	DM	62.6	0.7	basiliximab	mTORi/MMF/KS	No	Yes
6	m	GNF chr	51.5	2.9	basiliximab	CyA/MMF/KS	No	Yes
7	m	GNF chr	44.3	4.3	basiliximab	Tac/MMF/KS	No	Yes
8	m	ADPKD	50.3	0.8	basiliximab	Tac/mTORi/KS	No	Yes
9	m	TIN	42.7	4.7	basiliximab	Tac/MMF/KS	Yes	Yes
10	m	UNK	49.3	5.6	basiliximab	CyA/MMF/KS	No	Yes

PT ID: patient identifier; ESRD: end-stage renal disease; HD vintage: hemodialysis vintage; IS therapy: immunosuppressive therapy; TIN: tubulointerstitial nephritis; ADPKD: autosomal dominant polycystic kidney disease; VUR: vesicoureteral reflux; DM: diabetes mellitus; GNF chr: glomerulonephritis chronic; Tac: tacrolimus; MMF: mycophenolate mophetil; KS: corticosteroids; CyA: cyclosporine A, mTORi: mammalian target of rapamycin inhibitors.

**Table 2:** Clinical characteristics, indication, time, complications, and outcomes of ICD implantation.

PT ID	Age at ICD (Years)	Time of ICD Implantation after Tx (Years)	Cardiac Disease	Indication/ Prevention	Complications	Death	Time of Death after ICD (Years)	HD	Time of HD after ICD (Years)
1	63.4	−3.2	DCM	primary	0	No		Yes	8.9
2	62.3	16.2	ICM	primary	CVA	No		No	
3	69.4	8.2	ICM	primary	0	Yes	0.6	No	
4	62.5	7.1	ICM	primary	F-F bypass	No		Yes	4.5
5	69.1	6.5	ICM	primary	0	Yes	3.3	No	
6	53.4	1.8	DCM	primary	CVA	No		Yes	7.5
7	51.5	7.2	ICM	primary	0	Yes	4.8	No	
8	60.3	10.0	ICM	secondary	Pulmonary edema	No		No	
9	53.8	11.1	ICM	primary	0	Yes	1.8	No	
10	65.8	16.5	ICM	primary	0	No		No	

PT ID: patient identifier; DCM: dilatative cardiomyopathy; ICM: ischemic cardiomyopathy; CVA: cerebrovascular accident; F-F bypass: femoro-femoral bypass; HD: hemodialysis.

## Data Availability

Data supporting this study will be available upon request.
